# Cyberknife Radiosurgery and Concurrent Intrathecal Chemotherapy for Leptomeningeal Metastases: Case Report of Prolonged Survival of a HER-2+ Breast Cancer Patient Status-Post Craniospinal Irradiation

**DOI:** 10.7759/cureus.453

**Published:** 2016-01-07

**Authors:** Gregory Lekovic, Doniel Drazin, Albert C Mak, Marc S Schwartz

**Affiliations:** 1 Division of Neurosurgery, House Clinic; 2 Neurosurgery, Cedars-Sinai Medical Center; 3 Medical Director, Pasadena Cyberknife Center, Glendale Adventist Medical Center

**Keywords:** cyberknife radiosurgery, stereotactic radiosurgery, leptomeningeal disease, leptomeningeal metastases, intrathecal chemotherapy, radiation, breast cancer, neurosurgery, neurooncology

## Abstract

Leptomeningeal disease (LMD) from breast cancer is usually a rapidly fatal condition, with median overall survival reported to be 15 weeks. Conventional treatment for LMD includes craniospinal irradiation and intrathecal (IT) methotrexate. However, the role of stereotactic radiation for leptomeningeal disease remains poorly defined. This case report describes our experience using Cyberknife radiosurgery to treat a 49-year-old female with HER-2+ breast cancer and focal/nodular leptomeningeal metastases that were refractory to craniospinal irradiation and concurrent IT chemotherapy. This combined approach--i.e., craniospinal irradiation, IT chemotherapy, and Cyberknife Radiosurgery for local, recurrent metastases--resulted in survival of 46 months with controlled disease. Based on our experience with this patient, we believe further consideration of radiosurgery for LMD is warranted.

## Introduction

We present here a case report of a patient who had Cyberknife stereotactic radiosurgery (SRS) as a salvage treatment for focal, recurrent, leptomeningeal disease (LMD) status post craniospinal irradiation (CSI) and intrathecal trastuzumab (Herceptin). The appropriate role of focal treatments such as SRS for LMD is not well characterized. In particular, whether there is a benefit to SRS treatment for the patient who has received CSI and IT chemotherapy (or immunotherapy), but has nodular or focal recurrent and/ or residual disease in the leptomeninges, is unknown. Because LMD is thought to be a diffuse disease process, with tumor cells circulating in the CSF, there is little or no support for primary focal treatment such as radiosurgery (or surgery for that matter). This is in stark contradistinction to intraparenchymal brain metastases, which are commonly treated with radiosurgery as a primary treatment modality. 

Although the prognosis of patients with HER-2 positive (HER-2+) breast cancer has improved with the introduction of immunotherapy, specifically with the monoclonal antibody trastuzumab (Herceptin, Roche, Basel, Switzerland), leptomeningeal spread of breast cancer is still associated with very poor prognosis. Leptomeningeal metastases are thought to occur in about 5% of early-stage breast cancer patients, with no significantly effective treatment modality having been reported [1]. Scott et al. reviewed 36 studies reporting on LM metastases in breast cancer which showed a median overall survival of 15 weeks [1]. Of 851 patients reviewed, a majority (87%) received intrathecal (IT) methotrexate, which was associated with an increase in median survival to 18 weeks. These relatively dismal results with IT methotrexate have prompted oncologists to seek alternative approaches, including IT trastuzumab (Herceptin) [2-4].  The specific rationale for the use of IT Herceptin in our patient, as well as the specific dosing regimen of the same, has been previously published and is not the focus of this report [4]. 

We believe that Cyberknife SRS, in combination with systemic chemotherapy, CSI, and IT Herceptin, contributed to the prolonged survival of our patient, who lived for over 50 months after the diagnosis of LMD. We do not attribute her prolonged survival to the use of Cyberknife alone; rather, we contend that because of the significantly high-risk location of her nodular LMD lesions--especially one at the cervicomedullary junction--SRS was instrumental in controlling specific foci of disease, that if left untreated, would have precluded survival. We believe that further consideration for the use of SRS in the setting of LMD is therefore warranted. 

## Case presentation

A 49-year-old patient with a seven-year history of Her-2+ breast cancer and systemic metastases controlled with gemcitabine and trastuzumab (Herceptin, Roche, Basel, Switzerland) presented with a ten-day history of left-sided facial weakness and four months of left-sided hearing loss and severe imbalance. Magnetic resonance imaging (MRI) demonstrated punctate enhancement within the left internal auditory canal and a 6 x 5 mm lesion in Meckel’s cave. Given her history of metastatic breast cancer, the presumptive diagnosis of LM was made. Further imaging of the entire neuraxis was performed, revealing additional LM enhancement of the right S2 nerve root and dorsal surface of the cord at T5-T6. An additional new boney metastasis was noted at L2. IT methotrexate and craniospinal irradiation were recommended for treatment of her diffuse metastatic disease.

Fearing potential cognitive complications from craniospinal irradiation, the patient refused neuraxis irradiation and instead elected Cyberknife radiosurgery to treat the symptomatic lesions in her internal auditory canal (IAC) and Meckel’s cave. Both the IAC and Meckel’s cave lesions were treated with a prescription dose of 24 Gy to the 80% isodose line in three fractions of 8 Gy. The IAC tumor volume was 2,998 mm^3^ and the Meckel’s cave lesion 1,800 mm^3^. There was 97% and 98% coverage obtained of both tumor volumes, respectively. The patient was treated using a 6D skull tracking on a Robo-couch without complication.

The patient also refused IT methotrexate and was instead enrolled in an experimental protocol for IT trastuzumab. The protocol for the patient’s experimental IT Herceptin regimen has been published elsewhere [4]. Varying dosages of IT trastuzumab were given, starting at the lowest dose (5 mg weekly) in order to adequately assess safety and efficacy, and escalating to 50-80 mg IT per week. Systemic chemotherapy in addition to IT trastuzumab (Herceptin, Roche, Basel, Switzerland), was initiated after an uncomplicated placement of an Ommaya reservoir. Cytology was noted to be positive for malignant cells on CSF obtained during placement of the Ommaya reservoir.

### Follow-up treatment

Three months after the initial Cyberknife treatment, the patient stated she had significant improvement in her balance complaint and eye closure, but continued to suffer hearing loss. Her KPS was 90. MRI at that time demonstrated decreased enhancement of the leptomeninges in the IAC and Meckel’s cave, with linear enhancement of the left C1 nerve root now seen and a small (3mm) enhancing lesion in the left anterior cerebellum visualized. The overall imaging findings were felt to be consistent with LM carcinomatosis.

### Craniospinal radiation

Follow-up MRI six months after her initial Cyberknife treatment demonstrated an incomplete imaging response of the Meckel’s cave and IAC lesions with persistent enhancement; no progression of disease was noted. Unfortunately, the patient experienced distal progression of disease with increased enhancement seen at the cervicomedullary junction at C1-C2 (5 x 6 x 9 mm APxTVxCC) and left cerebellar hemisphere (9 x 8 x 6 mm APxTVxCC). In the thoracic spine, MRI demonstrated stable LM enhancement at T5-T6 and new enhancement at T7-T8. Lumbosacral spine MRI showed stable or reduced enhancement of the leptomeninges.

The patient was again referred for craniospinal irradiation to which she now agreed. The entire neuraxis was treated with a prescription dose of 3,000 cGy in fifteen treatment fractions followed by an additional 600 cGy boost to the spinal metastases. In response to these new metastases, the patient’s IT trastuzumab was also increased, escalating to a dose of 50-80 mg IT per week, from an initial weekly IT dose of 5 mg.

### Second Cyberknife treatment

**Figure 1:**
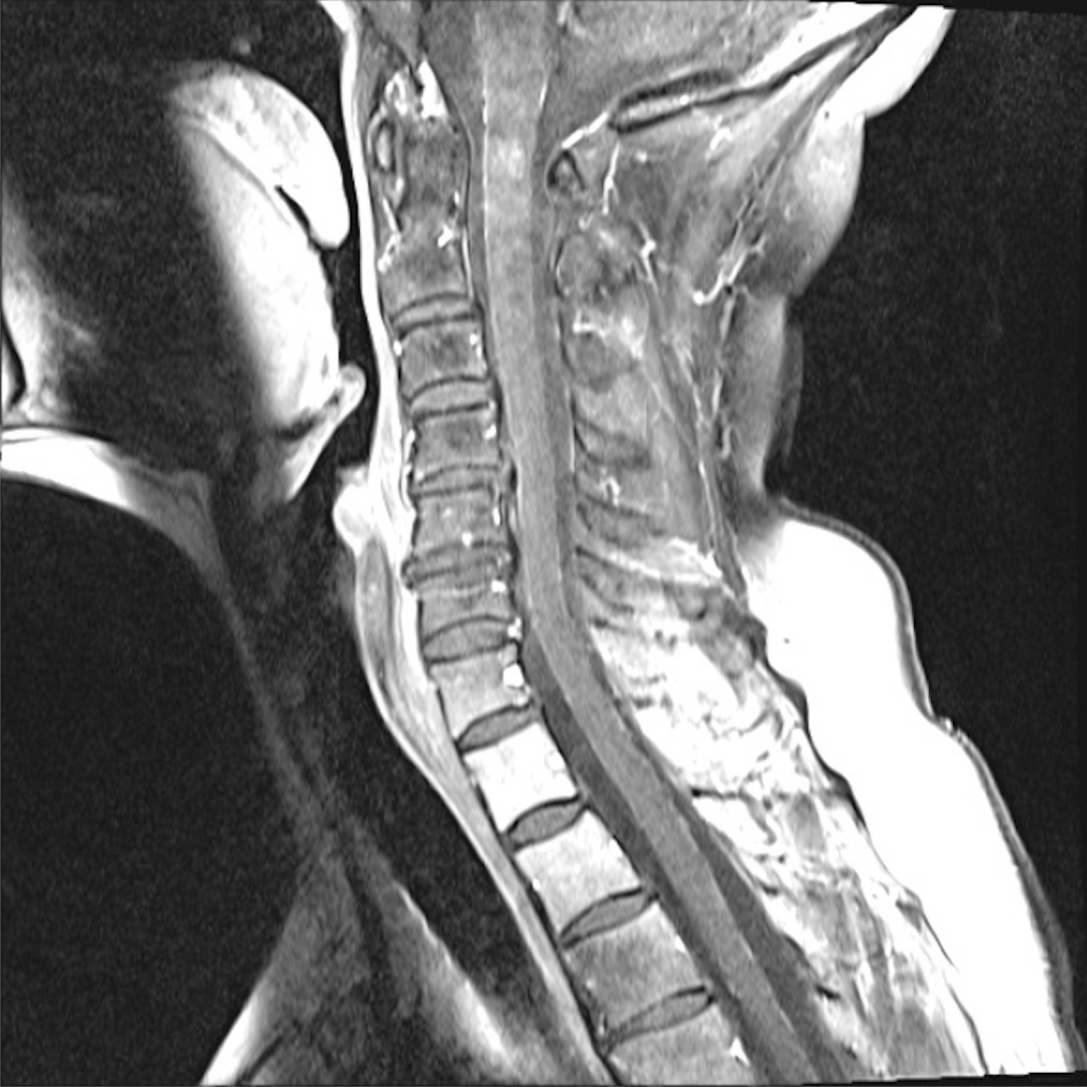
Cervical Spine MRI Sagittal MRI of the cervical spine demonstrated nodular contrast enhancement of the lesion at the cervicomedullary junction.

MRI imaging six months after completion of craniospinal irradiation demonstrated improvement in LM disease of the cerebellum and spine, however, increased nodularity of enhancement at C1-C2 was seen (Figure 1). At the same time, the patient became increasingly symptomatic with generalized weakness, unsteady gait, and left-sided ear and facial pain. The patient was subsequently offered Cyberknife radiosurgery to the cervicomedullary metastasis as a salvage treatment following failed craniospinal irradiation and IT trastuzumab. The prescription dose was 25 Gy in five fractions to the 77% isodose line; 96% coverage of the tumor volume was obtained. The maximum dose was 32.5 Gy. The treated tumor volume was 491 mm^3^. A total of 4,509 mm^3^ of spinal cord received a mean and maximum dose of 4.5 Gy and 29.3 Gy, respectively.

### Third Cyberknife treatment

Three months later (a total of 23 months after initial presentation with LM disease), the progressive nodular enhancement seen in the surface of the left cerebellar hemisphere (maximum dimension ~1.5 cm) was treated with Cyberknife radiosurgery. This tumor volume was 1,670 mm^3^; the prescription dose was 18 Gy at the 78% isodose line. Treatment was completed in a single fraction.

Throughout this follow-up period, the patient was continued on systemic chemotherapy as well as IT trastuzumab. The patient’s IT trastuzumab was delivered by Ommaya reservoir until she developed meningitis, mandating the Ommaya reservoir removal.  A new reservoir was placed, and the patient resumed IT chemotherapy one month later.

The patient was clinically stable and followed with MRI imaging of the neuraxis every three months; two years after the third Cyberknife treatment (40 months following initial presentation with LM disease), MRI demonstrated stable cerebellar, IAC, and cervicomedullary lesions with progression of enhancement in the left pterygopalatine fossa consistent with perineural spread of tumor from the left Meckel’s cave lesion. There was also noted diminished T2 signal in the cervicomedullary junction.

**Figure 2:**
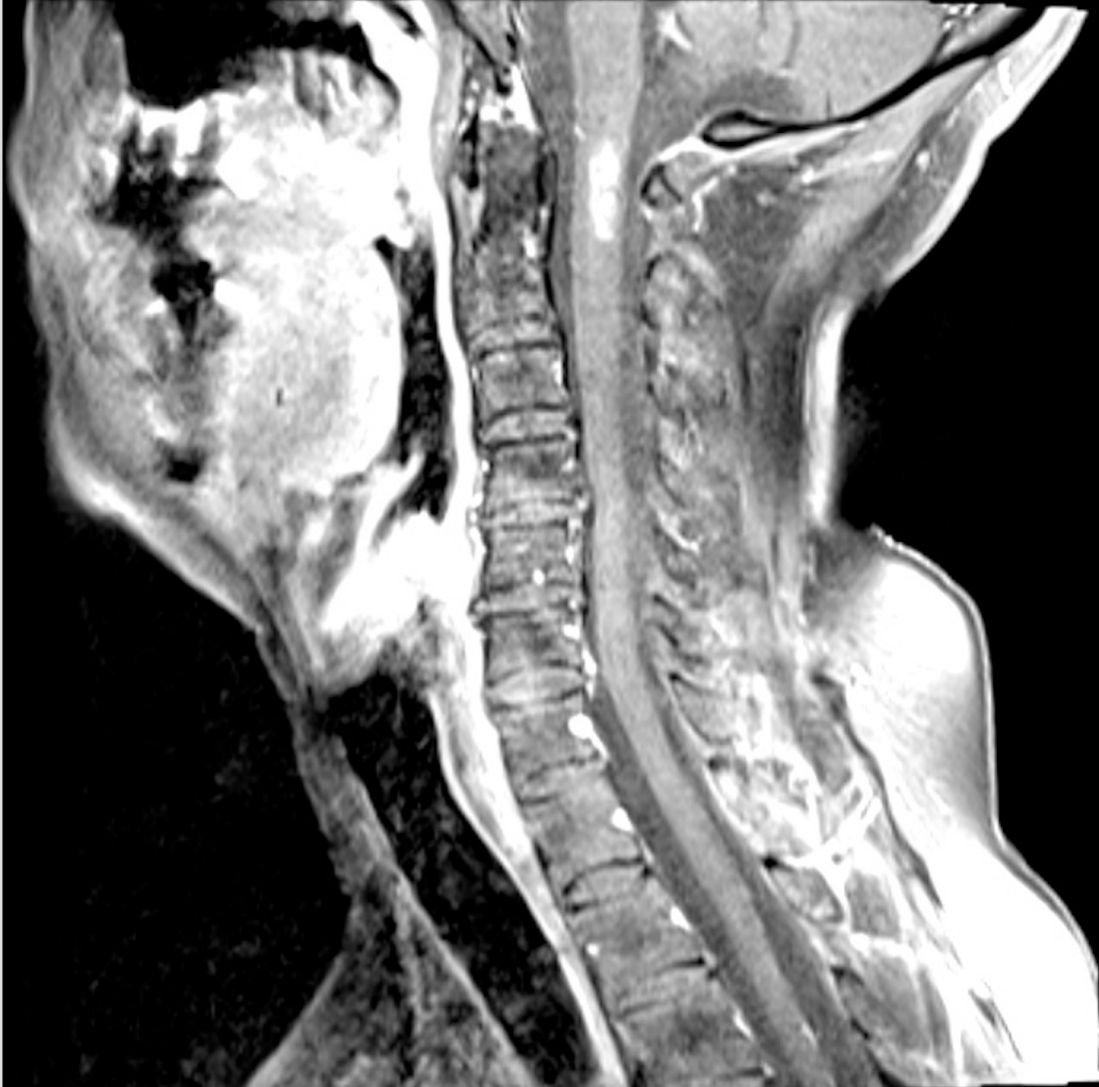
Cervical Spine MRI showing rapid enlargement Sagittal MRI of the cervical spine demonstrated substantial and rapid enlargement of the contrast enhancing lesion at the cervicomedullary junction.

Approximately three and a half years after her initial presentation, the patient became increasingly symptomatic with left-sided numbness, nausea and vomiting, and severe orthostatic blood pressure changes. Her performance status declined during this period, and she was no longer able to continue to exercise vigorously. She subsequently developed vocal cord paresis on the left as well. MRI of the cervical spine demonstrated substantial and rapid enlargement of the contrast enhancing lesion at the cervicomedullary junction, to a craniocaudal dimension of 22 mm (Figure 2). This finding was felt to be consistent with either local recurrence or treatment effect. The patient was not felt to be a candidate for surgery or further radiosurgery and was given high-dose dexamethasone for amelioration of her symptoms. The remainder of her neuraxis imaging remained stable.

### Fourth Cyberknife treatment

**Figure 3:**
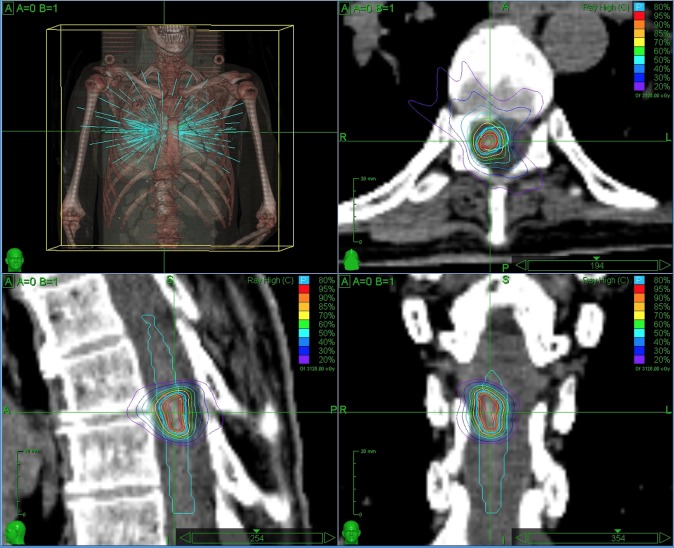
CyberKnife Treatment Plan (Fourth)

At 48 months after her initial presentation with LM metastases, the patient underwent a fourth Cyberknife treatment--this time to mid-thoracic LM disease at T7-T8 that had progressed on MRI, without cord compression or edema. The prescription dose was 2,500 cGy in five fractions to the 80% isodose line (peak dose 3,125 cGy) (Figure 3).

The cervicomedullary lesion continued to enlarge, with a maximal CC dimension of 32 mm on contemporaneous MRI of the cervical spine, and the patient continued to experience progressive neurologic deficits felt to be due to the cervicomedullary lesion. She became nonambulatory and complained of increased left-sided deficits that were refractory to steroids; she eventually succumbed, due to complications of respiratory failure, 52 months after her initial presentation with LM carcinomatosis.

### Imaging response

The patient was followed with serial MRI imaging every three months. Follow-up imaging demonstrated partial response of the IAC, anterior cerebellum, and cervicomedullary lesions and progression of disease involving the left Meckel’s cave and cavernous sinus. A one-year follow-up MRI after treatment of the cerebellar tumor showed stable intracranial and spinal disease, with the cervicomedullary tumor measuring (9 x 9 x 17 mm APxTVxCC); by eighteen months following the third Cyberknife treatment, MRI of the brain showed marked response with decreased enhancement of the cervicomedullary lesion to 6 x 7 x 9 mm. However, six months later, the patient had progression of the size of the enhancing lesion to over 24 mm.

Ultimately, the patient expired as a result of mass effect and edema of the caudal brainstem and rostral cervical spinal cord, e.g., on the nucleus ambiguous and/or other vagal nerve nuclei, leading to respiratory compromise and death.

## Discussion

This case report demonstrates the use of stereotactic radiosurgery as a salvage strategy for progression of disease after failed craniospinal radiation and IT trastuzumab. Although the first Cyberknife therapy was given as the patient’s initial therapy (i.e., before craniospinal radiation or IT chemotherapy), subsequent treatments were reserved for nodular/focal metastases after failure of craniospinal radiation and high-dose intrathecal trastuzumab to prevent progression of disease.

Stereotactic radiosurgery was initially well tolerated in spite of prior neuraxis radiation. It is unclear whether the patient experienced local recurrence after Cyberknife radiosurgery or whether she suffered from radiation necrosis at the cervicomedullary junction. We believe that the imaging progression of the patient’s cervicomedullary lesion reflected radiation effect rather than true local recurrence. The time-course of the patient’s enhancement and the association with profound T2 signal change may be interpreted as supporting evidence. The patient had prior craniospinal irradiation at 30 Gy, with a 6 Gy boost prior to Cyberknife. The volume of the spinal cord receiving therapeutic radiation dose was not insignificant. Because of the patient’s overall poor performance status, no attempt at biopsy and/or resection of radiation necrosis was made, however.

## Conclusions

This case report describes our experience treating a 49-year-old female with HER-2+ breast cancer and leptomeningeal disease using stereotactic radiosurgery. We conclude that Cyberknife radiosurgery for recurrent leptomeningeal disease, status post craniospinal radiation and intrathecal chemotherapy, contributed to this patient’s prolonged survival with stable disease for 46 months, longer than was anticipated given the extent of her disease progression and the limited resources currently available to treat LM metastases in the setting of HER-2+ breast cancer. We recommend that further experience with stereotactic radiosurgery for focal leptomeningeal metastases as a salvage treatment for patients with LM disease progression--after craniospinal radiation and intrathecal chemotherapy--is warranted.
